# Efficacy and Mechanism of Bazi Bushen Capsule on Skin Laxity: A Combination of Clinical and Network Pharmacology Study

**DOI:** 10.1111/jocd.70280

**Published:** 2025-06-30

**Authors:** Mo Zhao, Tuowei Li, Cuicui Cheng, Jun Mei, Fengqin Xu, Yuanbai Li, Yang Yang, Limin Yang, Jing Li, Xiaojie Zhang, Fangzhou Liu, Zhenhua Jia, Meng Hong

**Affiliations:** ^1^ Beijing University of Chinese Medicine Beijing China; ^2^ School of Food Science and Engineering South China University of Technology Guangzhou China; ^3^ Institute of Basic Research in Clinical Medicine China Academy of Chinese Medical Sciences Beijing China; ^4^ Institute of Geriatrics Xiyuan Hospital of China Academy of Chinese Medical Sciences Beijing China; ^5^ Institute of Information on Traditional Chinese Medicine China Academy of Chinese Medical Sciences Beijing China; ^6^ State Key Laboratory for Innovation and Transformation of Luobing Theory Hebei Yiling Hospital Hebei China; ^7^ EviSkin Testing Technology (Beijing) Co. Ltd. Beijing China; ^8^ Beijing Technology and Business University Beijing China

**Keywords:** Bazi Bushen Capsule, clinical research, mechanism, network pharmacology, skin laxity, traditional Chinese medicine

## Abstract

**Purpose:**

This study aims to explore the mechanism of Bazi Bushen Capsule (BZBS) in treating skin laxity by combining network pharmacology and clinical research.

**Methods:**

The active ingredients and potential drug targets of BZBS were obtained from TCMSP, TCMBANK, and SuperTCM databases. The potential disease targets of skin laxity were obtained from GeneCards, OMIM, and DisGeNET databases. The common core targets and key compounds were determined using Cytoscape software to construct the Drug Key Compound‐Target network and Protein–Protein Interaction network. The mechanism of BZBS in treating skin laxity was revealed by Gene Ontology and KEGG enrichment analysis. Subsequently, to further verify the analysis results, a prospective single‐group clinical trial was conducted, including 35 female volunteers with skin laxity. The planned study visits were initially scheduled for a 12‐week period. The volunteers' average depth of skin wrinkles, skin elasticity parameters, and skin moisture content were examined at 0 week before the experiment and 12 weeks after the experiment.

**Results:**

Network pharmacology shows that key compounds are quercetin, kaempferol, arachidonate, suchilactone, ammidin, deoxyharringtonine, sitosterol, mandenol, ethyl linolenate, stigmasterol, poriferast‐5‐en‐3beta‐ol, and cholesterol; core targets include AKT1, IL6, TP53, TNF, EGFR, TGFB1, JUN, MMP9, MTOR, and MMP2; the Relaxin, MAPK, PI3K‐Akt, JAK–STAT signaling pathways, and cellular senescence may be the main ways for BZBS in treating skin laxity. After BZBS treatment, the average wrinkling depth of the enrolled volunteers decreased, and the skin elasticity and moisture content increased.

**Conclusion:**

BZBS may treat skin laxity by repairing the mucosal barrier, regulating protein metabolism, and showing good therapeutic effects.

**Trial Registration:**

WHO‐recognized clinical trial registry: ChiCTR2200058262

## 1 Introduction

Skin laxity is a skin barrier damage manifestation characterized by a decrease in skin moisture, a decrease in elasticity, and deepening of wrinkles, caused by external factors such as sunlight ultraviolet rays and internal factors such as aging, heredity, and other factors. Its pathogenesis is unknown, and it is related to inflammatory responses, oxidative stress injury, cell autophagy, matrix metalloproteinase expression, collagen synthesis, DNA damage, and melanin synthesis [1, 2]. Modern medicine often uses chemical peeling and laser treatment, daily use of antioxidants to protect the skin, and sunscreen products to reduce skin exposure.

Bazi Bushen Capsule (BZBS) is a traditional Chinese medicine product made from 16 natural herbs, mainly used to treat knee and hip pain, dizziness, tinnitus, fatigue, forgetfulness, and lethargy caused by insufficiency of kidney yang. Our previous studies have found that this medicine has certain therapeutic effects on premature aging [3], and animal experiments have shown that it can improve cardiac aging [4], restore the damaged intestinal barrier [5], alleviate atherosclerosis and cognitive deficits [6, 7], but the effects on the skin are unknown.

**FIGURE 1:**
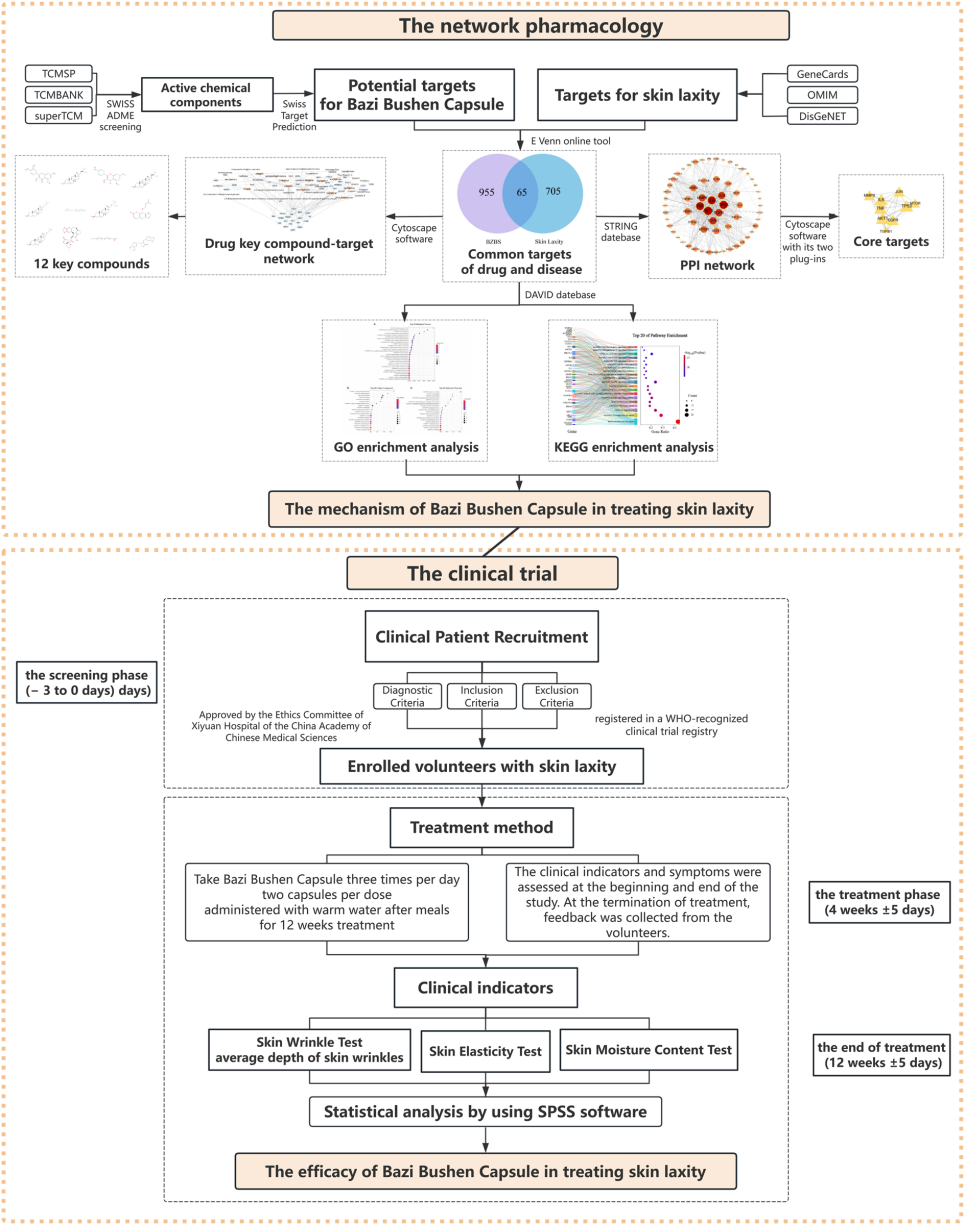
Flowchart of the study. Our research is divided into two parts. The dashed boxes above explain the ideas of network pharmacology research, and the research types and objectives are highlighted by the orange solid boxes. The dashed boxes below explain the process of clinical research, and the solid orange boxes highlight the types and objectives of the research.

Network pharmacology is a subdiscipline of pharmacology that uses network methods to analyze the “multicomponent, multitarget, multipathway” synergistic action relationships between drugs and diseases and targets. This method can help to understand the multicomponent synergistic enhancement mechanism of traditional Chinese medicine in treating diseases at the systemic level and molecular level [8], and further it can combined with clinical and experimental studies to prove the effectiveness of traditional Chinese medicine, which is currently an effective research paradigm for transforming traditional Chinese medicine from empirical medicine to evidence‐based medicine. This study aims to explore the mechanism of BZBS in treating skin laxity using network pharmacology research methods and validate it in a prospective single‐group clinical study. The flowchart of this study is shown in Figure 1.

## 2 Materials and Methods

### 2.1 Active Chemical Components Screening

The chemical components of each herb in BZBS were obtained from the TCMSP (http://tcmsp‐e.com/), TCMBANK (http://www.tcmip.cn/TCMBank/), and SuperTCM (https://bioinf‐applied.charite.de/supertcm/). Screening of active compounds was conducted based on Absorption, Distribution, Metabolism, and Excretion (ADME) parameters. For the TCMSP platform, oral bioavailability (OB) ≥ 30% and drug‐likeness (DL) ≥ 0.18 were set as the screening conditions, in accordance with the database's usage guidelines [9]. Additionally, for SWISS ADME, gastrointestinal absorption (GI absorption) was classified as “High,” and at least 2 out of 5 DL filters (Lipinski, Ghose, Veber, Egan, Muegge) had to return a “Yes” prediction [10].

### 2.2 Targets Screening of Potential Active Compounds

The targets prediction for BZBS was performed using Swiss Target Prediction (http://www.swisstargetprediction.ch/) [11], by inputting the canonical SMILES into SMILES string(s) [12, 13], with the organism selected as “ *Homo sapiens* .” The compound targets having no relationship with the compounds were deleted. Swiss is a free public resource to explore interactions between chemicals and gene targets. The potential targets are ranked by probability from high to low. Gene information, including gene ID, name, and organism, was identified using the UniProt database (https://www.uniprot.org/).

### 2.3 Targets Relevant to Skin Laxity

The GeneCards database (https://www.genecards.org/), OMIM database (https://omim.org/), and DisGeNET database (https://disgenet.cn/) were searched for genes related to skin laxity using “cutis laxa,” “skin laxity,” and “skin sagging” as the search terms [14, 15, 16]. The gene data related to skin laxity from the three databases were normalized by removing duplicates and standardizing names.

### 2.4 Construction of Drug Key Compound‐Target Network

Using the online tool E Venn (http://www.ehbio.com/test/venn/#/), the potential intersection targets of BZBS in the treatment of skin laxity were obtained [17]. The drug key compound‐target network was built by importing related common targets and each potential active compound with a relatively proportion derived from each herb in the BZBS into Cytoscape 3.10.0 software. The top 12 key compounds were found and listed eventually.

### 2.5 Construction of Protein–Protein Interaction Network for Common Targets

The common targets were imported into the STRING database (https://cn.string‐db.org/) [18] to construct a protein–protein interaction (PPI) network. The unconnected nodes were removed, and the minimum protein interaction confidence was set as 0.400 to generate the PPI network. TSV format results were downloaded and imported into Cytoscape 3.10.0 software, and the core targets were screened by analyze plug‐in, including cytoNCA and cytoHubba.

### 2.6 Gene Ontology and KEGG Enrichment Analysis

The common targets of BZBS and skin laxity were uploaded to the DAVID database (https://davidbioinformatics.nih.gov/summary.jsp) [19], and the species was set as “Homo” Gene Ontology (GO) and KEGG enrichment analysis were performed by sapiens, and enrichment results with *p* value < 0.01 were screened out to obtain enrichment and closely related biological processes and signaling pathways. With the help of the online tool WeiShengXin platform (https://www.bioinformatics.com.cn/), the results were visualized by bubble map and enriched Sanji bubble map to further understand the Biological Process (BP), Cellular Component (CC), Molecular Function (MF), and key pathways of BZBS in the treatment of skin laxity.

### 2.7 Clinical Studies

#### 2.7.1 Settings

This study is a prospective single‐group clinical trial aimed at evaluating the effectiveness of BZBS in improving skin laxity symptoms in women. The study was conducted at Xiyuan Hospital and recruited a total of 35 female volunteers aged 30–50. All enrolled volunteers met the diagnostic, inclusion, and exclusion criteria. They were informed of the purpose and procedures of the study and provided written informed consent before participating in the study.

##### 2.7.1.1 Diagnostic Criteria

Volunteers recruited were diagnosed by experienced TCM physicians and met the TCM diagnostic criteria for Kidney Essence Deficiency, confirmed through the Frailty Assessment Scale.

##### 2.7.1.2 Inclusion Criteria

Volunteers were included in the study if they satisfied the following criteria: (1) TCM diagnostic criteria for Kidney Essence Deficiency, with a Frailty Assessment Scale score ≥ 10; (2) female, aged 30–50 years; (3) had obvious symptoms of dry lines, fine lines, skin laxity, sagging, and had persisted for at least 6 months; (4) understood and were willing to comply with the study protocol and were able to provide written informed consent.

##### 2.7.1.3 Exclusion Criteria

Volunteers were excluded from the study if they: (1) had skin allergy; (2) had received radiofrequency or laser rhytidectomy within 6 months; (3) had received facial soft tissue filler injection, botulinum toxin injection, or other long‐term facial maintenance treatment within 1 year; (4) were pregnant, breastfeeding, or planning pregnancy; (5) were long‐term outdoor workers; (6) had severe liver or kidney dysfunction, malignant tumors, or other conditions affecting safety; (7) had severe mental disorders or cognitive impairment; (8) had been diagnosed with TCM syndromes such as Liver Yang Hyperactivity, Excess Heat, Damp‐Heat, or Yin‐deficiency‐heat syndrome; (9) had used medications or topical treatments affecting skin tightness and moisture content within the past 6 months; (10) had participated in other clinical trials within the last 2 months.

#### 2.7.2 Treatment Method

All enrolled volunteers were administered BZBS (Approval Number: B20020585) provided by Shijiazhuang Yiling Pharmaceutical Co. Ltd. Volunteers took the capsules three times per day, with two capsules per dose (0.4 g per capsule), and administered with warm water after meals.

#### 2.7.3 Volunteers Management

The planned study visits were initially scheduled for a 12‐week period, with three visits during the screening phase (−3 to 0 days), the treatment phase (4 weeks ± 5 days), and the end of treatment (12 weeks ± 5 days). The efficacy and safety of the drug were assessed at the beginning and end of the study. At the termination of treatment, feedback was collected from the volunteers, and changes in their symptoms were recorded. The treatment process strictly followed the clinical study protocol to ensure the accuracy and completeness of the data.

#### 2.7.4 Clinical Cure

The clinical evaluation was examined at 0 week before the experiment and 12 weeks after the experiment, involving: (1) the average depth of skin wrinkles obtained by EvaSKIN; (2) skin elasticity parameters obtained by skin elasticity test Cutometer MPA580; and (3) skin moisture content obtained by Corneometer CM825. All tests were conducted by trained technicians to ensure consistency and accuracy. The testing environment was controlled at a temperature of 20°C–22°C and humidity of 40%–60%. All equipment was calibrated before each test to ensure data reliability.

##### 2.7.4.1 Skin Wrinkle Test

It was done for all the volunteers to obtain the average depth of skin wrinkles by EvaSKIN (EOTECH, France). The digital optical 3D image analyzer EvaSKIN is a noncontact 3D image analysis system based on digital microscopic fringe projection technology, which is used to capture the morphological changes of the skin surface. The system generates a digital three‐dimensional image of the skin surface by recording and analyzing the bending deformation of the straightened light projected on the skin, and then quantifies parameters such as wrinkle depth.

##### 2.7.4.2 Skin Elasticity Test

It was done for all the volunteers to obtain the skin elasticity parameters including R2, R5, R7 by Cutometer MPA580 (Courage and Khazaka, Germany). The Cutometer is a noncontact optical testing system, which tests the deformation and recovery ability of the skin under negative pressure to evaluate the elastic and viscoelastic properties of the skin.

##### 2.7.4.3 Skin Moisture Content Test

It was done for all the volunteers to obtain the skin moisture content by Corneometer CM825 (Courage and Khazaka, Germany). It is based on the principle of capacitance and the dielectric constant of water. When the moisture content of the skin changes, the capacitance value of the skin also changes. Therefore, the water content of the skin surface can be analyzed by measuring the capacitance value of the skin. The readings reflect changes in the moisture on the surface of the skin.

#### 2.7.5 Statistical Analysis

Statistical analysis was performed using SPSS in this study. Continuous variables were described and tested by different methods according to whether they conformed to normal distribution. The *t‐*test was used if homogeneity of variance was satisfied, and the Wilcoxon signed‐rank test was used if not. The significant value was set at *p* value < 0.05.

## 3 Results

### 3.1 Network Pharmacology Analysis

#### 3.1.1 Drug Key Compound‐Target Network Analysis

**FIGURE 2:**
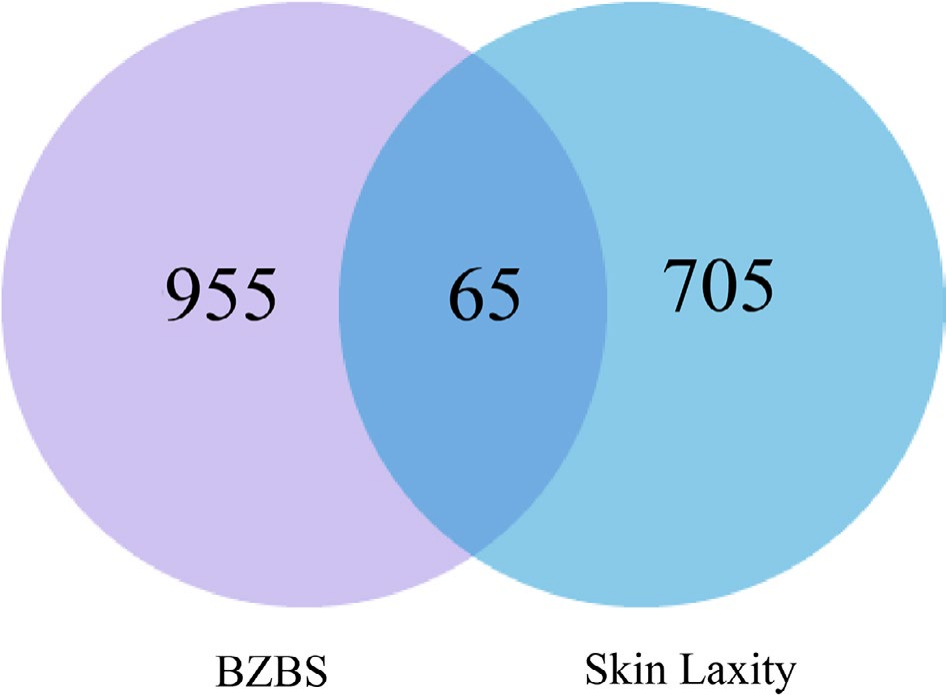
E Venn diagram of BZBS and skin laxity. Purple is the drug targets. Blue is the disease targets. In the middle are the common targets.

A total of 1020 drug targets and 770 disease targets were input into online tool E Venn, and 65 common targets were obtained, as shown in Figure 2.

**FIGURE 3:**
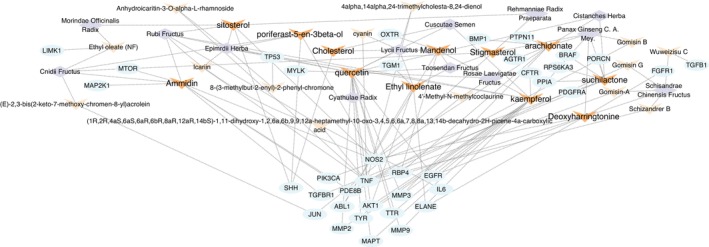
Drug Key Compound‐Target network. Light orange arrows indicate the active compounds of each herb from BZBS, orange arrows highlight the top 12 key compounds, light blue circles indicate the common targets, light purple diamonds indicate the herbs of BZBS, and dashed lines indicate the connections between the herbs, active compounds, and common targets.

**FIGURE 4:**
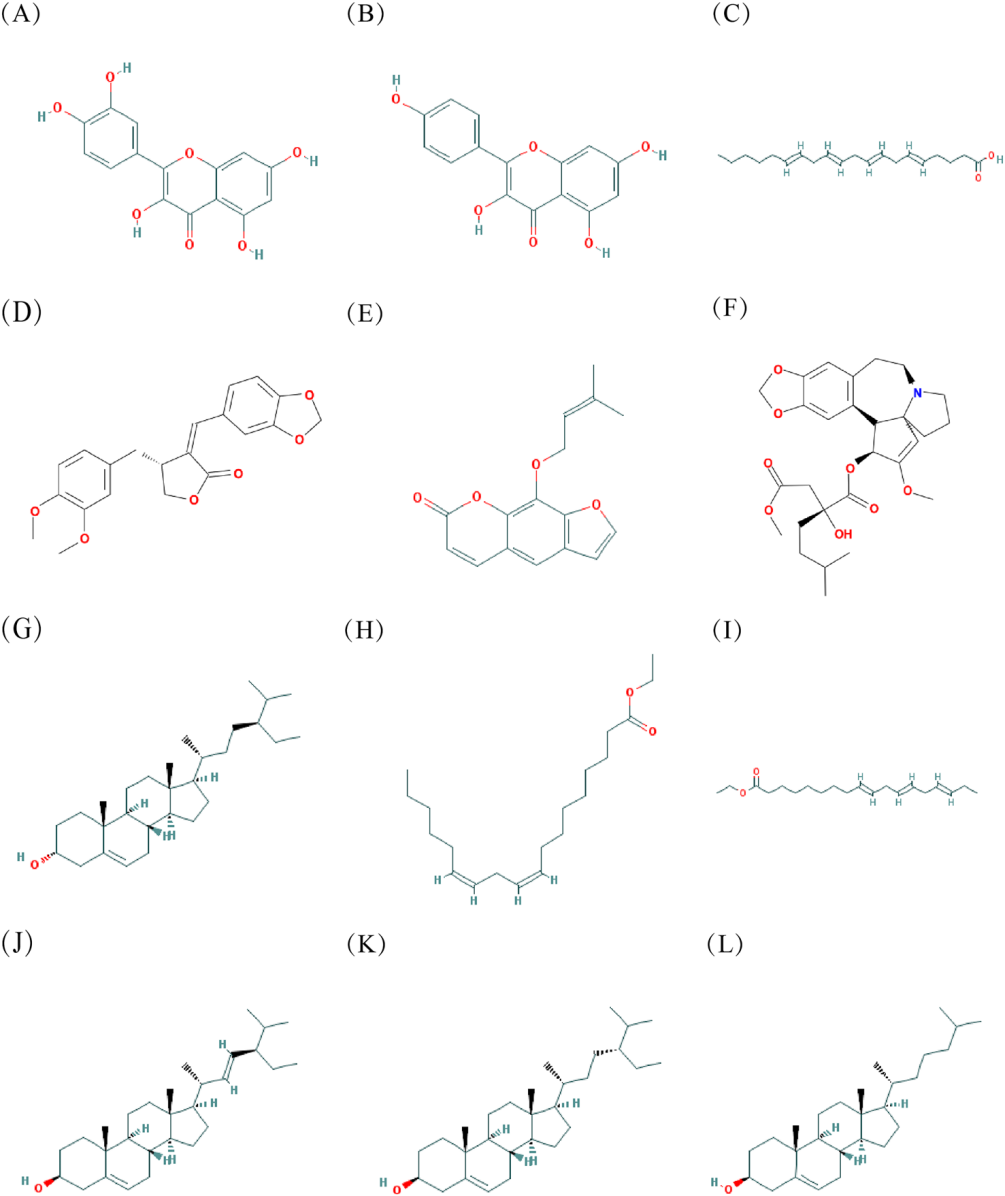
The top 12 key compounds. (A) The structure of quercetin. (B) The structure of kaempferol. (C) The structure of arachidonate. (D) The structure of suchilactone. (E) The structure of ammidin. (F) The structure of deoxyharringtonine. (G) The structure of sitosterol. (H) The structure of mandenol. (I) The structure of ethyl linolenate. (J) The structure of stigmasterol. (K) The structure of poriferast‐5‐en‐3beta‐ol. (L) The structure of cholesterol.

In order to describe the relationship between the common targets and active compounds from each herb and to screen out the key compounds, a drug key compound‐target network with 77 nodes and 126 edges was constructed. We removed two unrelated common targets. The remaining 63 related common targets and each active compound with a relatively proportion derived from each herb in the BZBS were imported into Cytoscape 3.10.0 software to illustrate the network, as shown in Figure 3. The key compounds were ranked according to the closeness of node connectivity calculated by the cytoNCA plug‐in in the network. The first 12 were respectively quercetin, kaempferol, arachidonate, suchilactone, ammidin, deoxyharringtonine, sitosterol, mandenol, ethyl linolenate, stigmasterol, poriferast‐5‐en‐3beta‐ol, and cholesterol, as shown in Table 1 and Figure 4.

**TABLE 1:** Specific information on the top 12 key compounds.

Molecule ID	Molecule name	OB (%)	DL	Betweeness	Closeness	Herb
MOL000098	Quercetin	46.43	0.28	1476.37	0.41	Cyathulae Radix, Rubi Fructus, Lycii Fructus, Rosae Laevigatae Fructus, Cuscutae Semen, Epimrdii Herba, Cistanches Herba, Toosendan Fructus
MOL000422	Kaempferol	41.88	0.24	977.76	0.39	Rubi Fructus, Rosae Laevigatae Fructus, Panax Ginseng C. A. Mey., Cuscutae Semen, Epimrdii Herba
MOL005320	Arachidonate	45.57	0.2	632.13	0.33	Panax Ginseng C. A. Mey., Cistanches Herba
MOL005384	Suchilactone	57.52	0.56	843.14	0.31	Panax Ginseng C. A. Mey., Cistanches Herba
MOL001941	Ammidin	34.55	0.22	721.53	0.34	Rubi Fructus, Cnidii Fructus
MOL005317	Deoxyharringtonine	39.27	0.81	819.49	0.34	Panax Ginseng C. A. Mey., Schisandrae Chinensis Fructus
MOL000359	Sitosterol	36.91	0.75	402.83	0.32	Epimrdii Herba, Morindae Officinalis Radix, Rehmanniae Radix Praeparata, Rubi Fructus
MOL001494	Mandenol	42	0.19	323.61	0.29	Lycii Fructus, Rosae Laevigatae Fructus, Toosendan Fructus
MOL001495	Ethyl linolenate	46.1	0.2	305.82	0.29	Toosendan Fructus, Lycii Fructus
MOL000449	Stigmasterol	43.41	0.76	355.30	0.34	Toosendan Fructus, Rehmanniae Radix Praeparata, Panax Ginseng C. A. Mey., Cnidii Fructus
MOL001771	Poriferast‐5‐en‐3beta‐ol	36.91	0.75	224.61	0.32	Cnidii Fructus, Epimrdii Herba
MOL000953	Cholesterol	37.87	0.68	55.32	0.28	Lycii Fructus, Cuscutae Semen

#### 3.1.2 PPI Network Analysis

**FIGURE 5:**
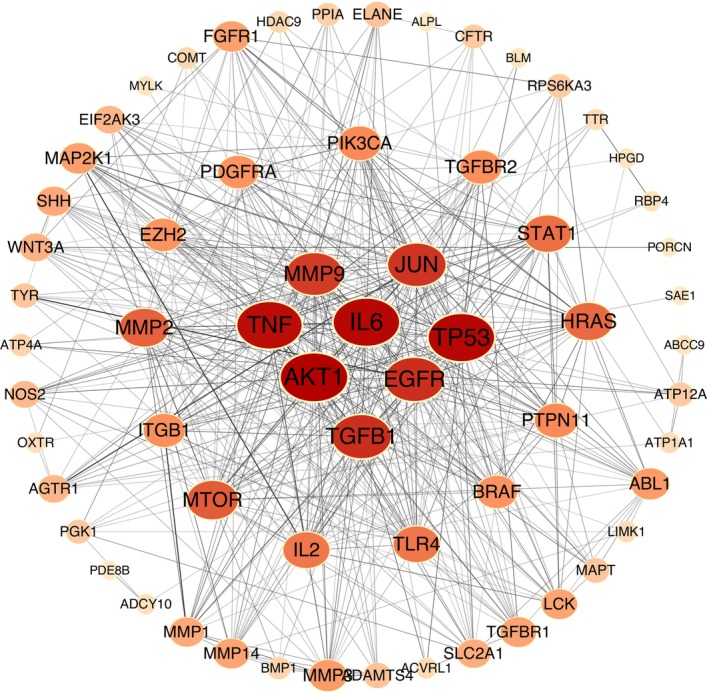
The PPI network and the targets obtained by the plug‐in cytoNCA operation.

PPI network analysis can visually express the main functions of proteins, the interaction between proteins, and the degree of correlation. With the help of the Cytoscape software, we conducted a PPI network analysis to explore the relationships between different targets and to find the core targets (Figure 5). Sixty‐five common targets were imported into STRING, and the species selected was *Homo sapiens* with a confidence level > 0.400. There were 65 nodes and 498 edges in the network. The average node degree value was 15.3.

**FIGURE 6:**
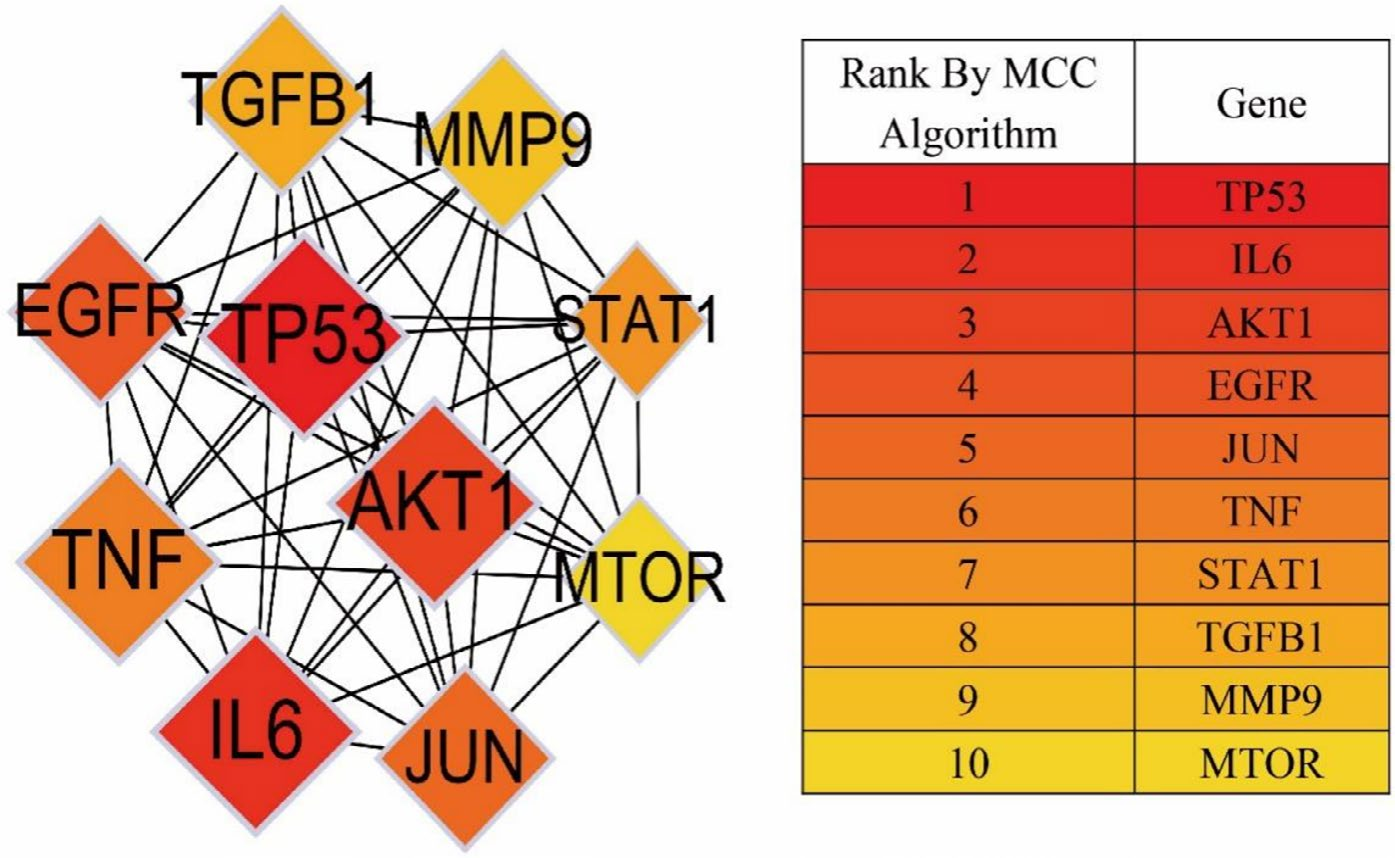
The targets obtained by the plug‐in cytoHubba operation.

**FIGURE 7:**
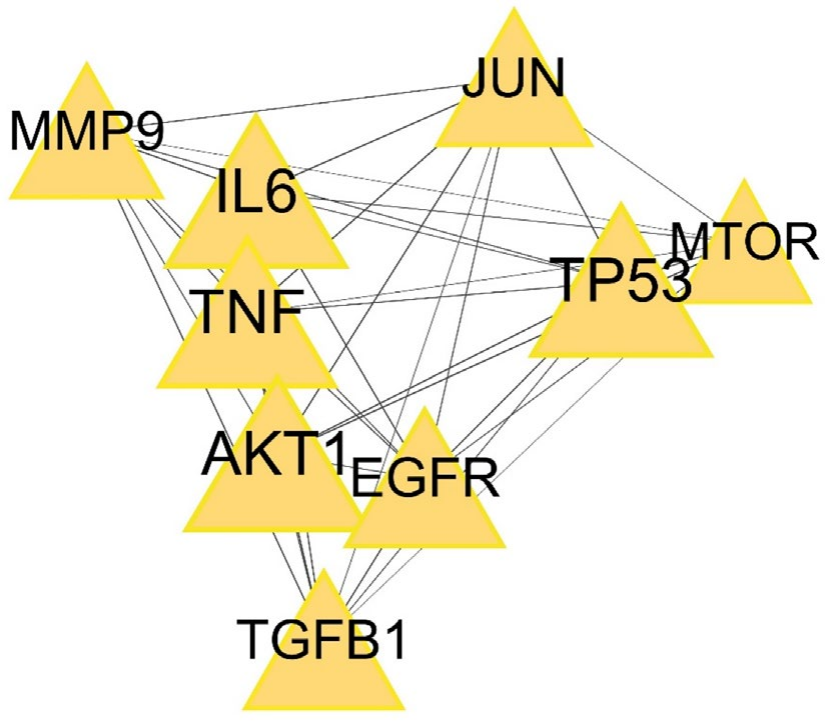
The nine core targets obtained by intersection of the operation results of the two plug‐ins cytoNCA and cytoHubba.

We exported the PPI network TSV file from the String software and used Cytoscape 3.10.0 with its two plug‐ins to illustrate the core targets. The cytoNCA plug‐in generated Figure 4 by calculating the betweenness, closeness, and degree of nodes in the network, setting the betweenness threshold to the median of 13.8, the closeness threshold to the median of 0.53, and the degree threshold to the median of 14. In the network, the circle represented each common target, its size, color depth, and edge thickness represented their degree values, the line represented the connections between them, and the color depth of the line represented the closeness of the connection. Therefore, the top 10 targets ranked according to the degree value were respectively obtained: AKT1, IL6, TP53, TNF, EGFR, TGFB1, JUN, MMP9, MTOR, and MMP2. The cytoHubba plug‐in drew Figure 6 by using the MCC algorithm and identified the top 10 targets in the network. The diamonds in the network and the table were colored from dark to light to represent the ranking of targets. The lines represented the connections between the targets. We took the intersection of the operation results of the two plug‐ins and obtained nine core targets: AKT1, IL6, TP53, TNF, EGFR, TGFB1, JUN, MMP9, MTOR, as shown in Figure 7 and Table 2. The yellow triangles in the network symbolized the core targets, and the lines played bridge‐spanning roles.

**TABLE 2:** The nine core targets.

Target	Description	Degree	Betweeness	Closeness	Score
AKT1	AKT serine/threonine kinase 1	44	385.0871	0.7654321	3.88E+10
IL6	Interleukin 6	43	337.51065	0.75609756	3.89E+10
TP53	Tumor protein p53	43	404.60046	0.74698794	3.89E+10
TNF	Tumor necrosis factor	42	291.98392	0.74698794	3.73E+10
EGFR	Tumor necrosis factor	37	203.935	0.6966292	3.89E+10
TGFB1	Transforming growth factor beta 1	37	210.96762	0.6888889	3.57E+10
JUN	Jun proto‐oncogene, AP‐1 transcription factor subunit	36	147.36777	0.6888889	3.85E+10
MMP9	Matrix metallopeptidase 9	35	118.64913	0.6813187	3.52E+10
MTOR	Mechanistic target of rapamycin kinase	30	96.31097	0.6458333	3.51E+10
Target	Description	Degree	Betweeness	Closeness	Score

#### 3.1.3 GO and KEGG Enrichment Analysis

The 63 connected key targets identified by the PPI network on the DAVID database were subjected to GO function and KEGG pathway enrichment analysis. A *p* value of < 0.01 was set for species “ *Homo sapiens* .” 111 KEGG pathways and a total of 1820 GO analysis terms were obtained, including 164 BP, 25 CC, and 34 MF.

#### 3.1.4 GO Function Enrichment Analysis

**FIGURE 8:**
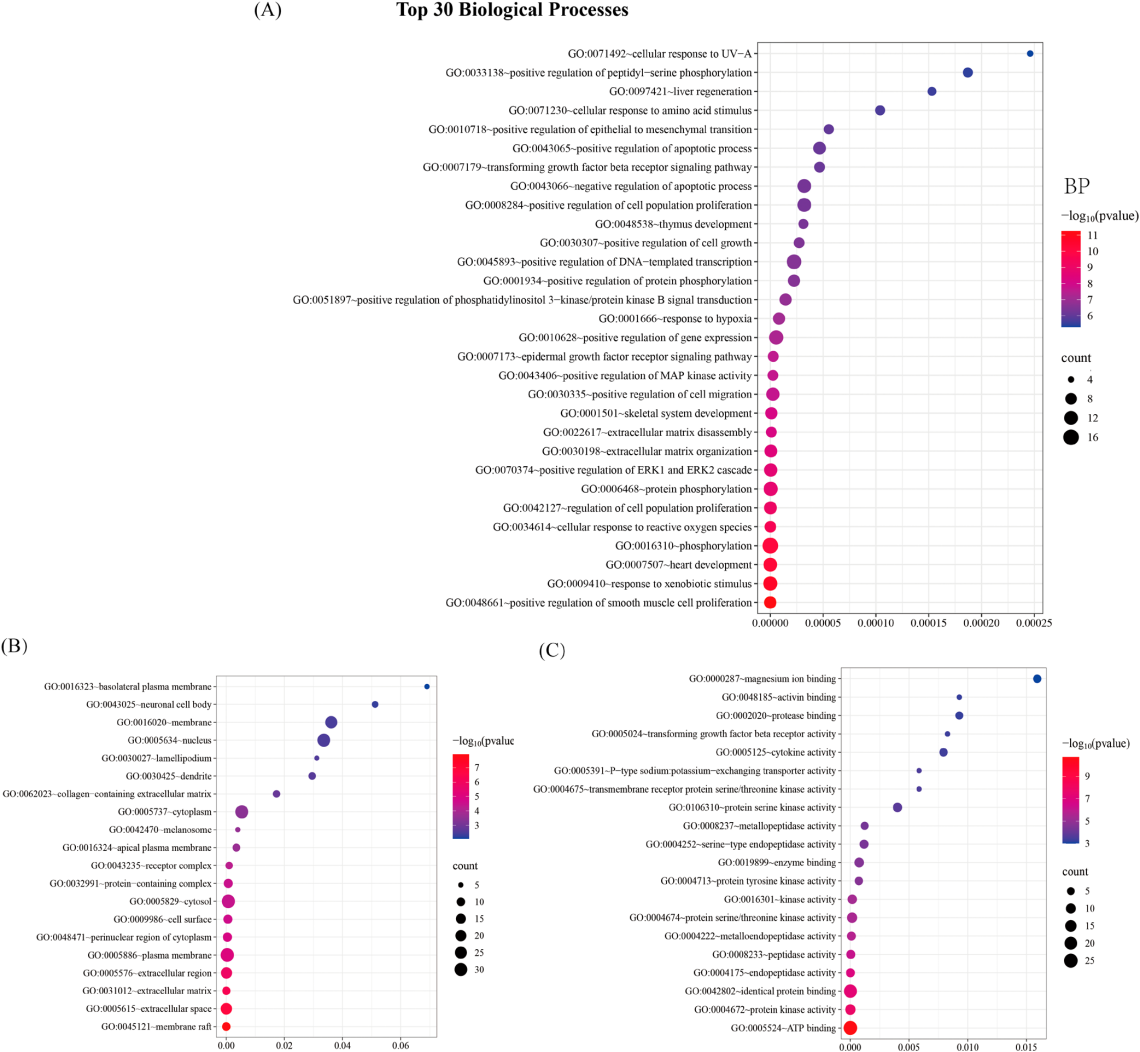
GO enriched analysis. (A) The top 30 *p* value results of biological process. (B) The top 20 *p* value results of cellular component. (C) The top 20 *p* value results of molecular function.

Take the top 30 *p* value results of BP, the top 20 results of CC and MF, and use the WeiShengXin platform to create a GO bubble map (Figure 8). The bubble size represents the number of enriched genes, and the color from purple to red represents the decreasing *p* value. The BP are mainly concentrated on positive regulation of smooth muscle cell proliferation, response to xenobiotic stimulus, heart development, phosphorylation, and cellular response to reactive oxygen species. The CC are mainly concentrated on membrane raft, extracellular space, extracellular matrix, extracellular region, and plasma membrane. The MF are mainly concentrated on ATP binding, protein kinase activity, identical protein binding, endopeptidase activity, and peptidase activity.

#### 3.1.5 KEGG Function Enrichment Analysis

**FIGURE 9:**
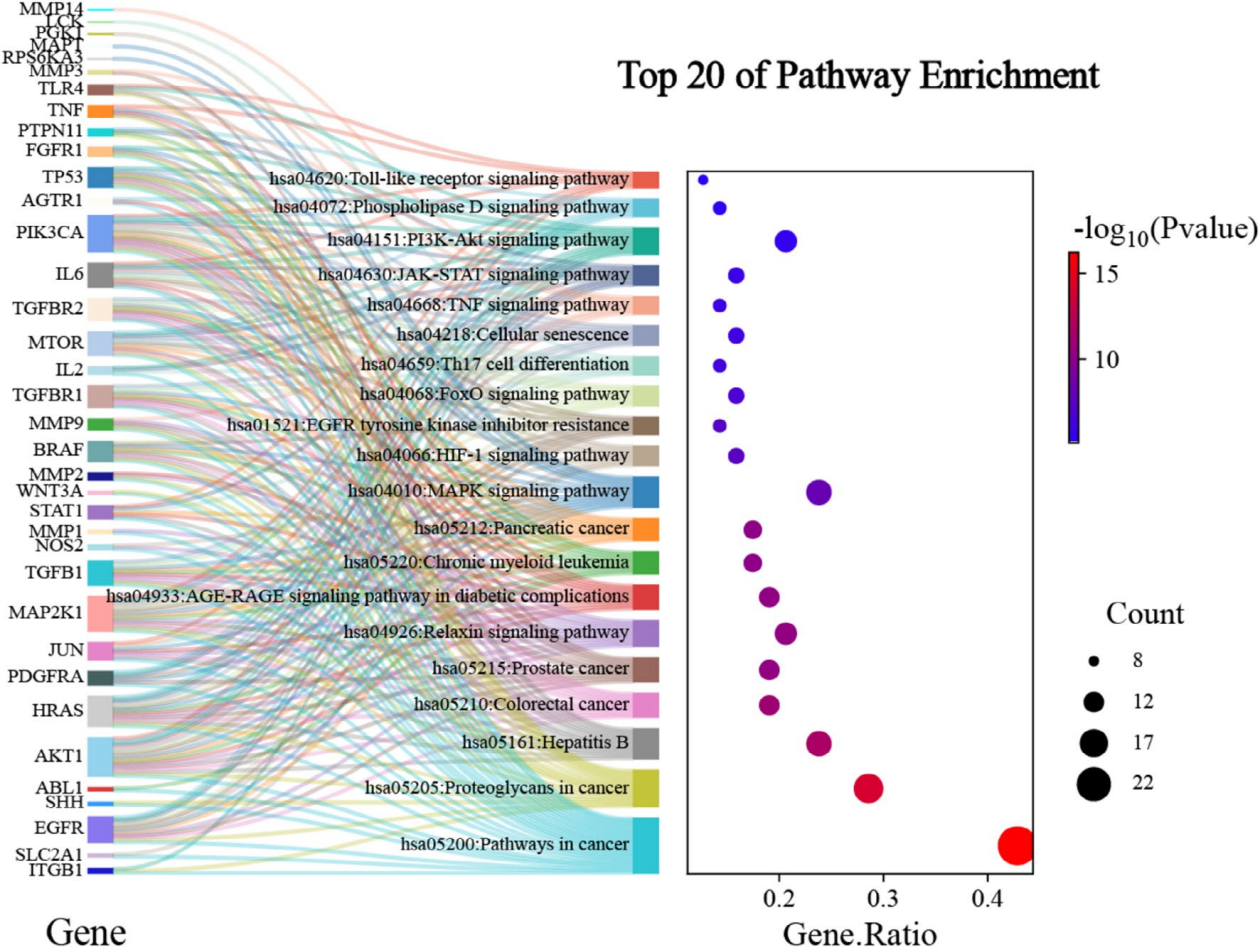
The top 20 *p* value results of KEGG enriched analysis.

Take the top 20 *p* value results of KEGG pathways, and use the WeiShengXin platform to create a KEGG Sanji enriched bubble map (Figure 9). The first left column of colored squares represents different gene targets, the second column of colored squares represents different pathways, the size of the colored squares all represents the number of enriched gene targets, and the line of the corresponding color symbolizes the connection between each pathway and enriched gene target. In the third column of white squares, the bubble size represents the number of enriched gene targets, and from purple to red represents the decrease in *p* values. The KEGG pathways are mainly enriched in pathways associated with cancer, including colorectal cancer, prostate cancer, pancreatic cancer, and proteoglycans in cancer. The results showed that BZBS may play an important role in the treatment of skin laxity through the Relaxin signaling pathway, mitogen‐activated protein kinase (MAPK) signaling pathway, phosphoinositide 3‐kinase (P13K)‐protein kinase B (Akt) signaling pathway, Janus tyrosine kinase (JAK)‐signal transducer and activator of transcription (STAT) signaling pathway, and cellular senescence, which are related to collagen synthesis and inflammatory response.

### 3.2 Clinical Study

According to the diagnosis, inclusion and exclusion criteria, a total of 35 female volunteers with saggy skin were enrolled in this study. The skin wrinkles, skin elasticity, and skin moisture were significantly improved after 12 weeks of BZBS treatment.

#### 3.2.1 Average Depth of Skin Wrinkles

The average wrinkling depth of the enrolled volunteers was 61.46 ± 22.79 μm at the time of enrollment, and after 12 weeks of BZBS treatment, the wrinkling was significantly lighter, with a depth of only 56.43 ± 22.32 μm, and the difference was statistically significant (*p* value < 0.001), shown as Table 3.

**TABLE 3:** Comparison of average depth of skin wrinkles before and after treatment ($\bar{X}$ ± *S*).

	Detection time	Average depth of skin wrinkles (μm)	*p*
Prior‐treatment	0 week	61.46 ± 22.79	< 0.001***
Post‐treatment	12 week	56.43 ± 22.32***	

*p < 0.05, **p < 0.01, ***p < 0.001.

#### 3.2.2 Skin Elasticity

Skin elasticity test Cutometer MPA580 was used to evaluate skin elasticity in three dimensions, namely R2, R5, and R7. After 12 weeks of BZBS treatment, R5 increased from 0.57 ± 0.07 before treatment to 0.61 ± 0.09 after treatment (*p* value < 0.05). R2 and R7 also increased slightly, but the difference was not statistically significant (Table 4).

**TABLE 4:** Comparison of skin elasticity before and after treatment ($\bar{X}$ ± *S*).

Skin elasticity	Detection time		*p*
	Prior‐treatment (0 week)	Post‐treatment (12 week)	
R2	0.58 ± 0.07	0.59 ± 0.07	0.449
R5	0.57 ± 0.07	0.61 ± 0.09*	0.026*
R7	0.38 ± 0.05	0.40 ± 0.05	0.105

*p < 0.05, **p < 0.01, ***p < 0.001.

#### 3.2.3 Skin Moisture Content

The skin of the enrolled volunteers was generally in a state of dehydration, and the water content was only 66.78 ± 10.35 C.U. before treatment. After 12 weeks of uninterrupted administration of BZBS, the skin condition was improved, and the water content increased to 70.03 ± 9.91 C.U., and the difference was statistically significant (*p* value < 0.05), shown as Table 5.

**TABLE 5:** Comparison of skin moisture content before and after treatment ($\bar{X}$ ± *S*).

	Detection time	Skin moisture content (C.U.)	*p*
Prior‐treatment	0 week	66.78 ± 10.35	0.038*
Post‐treatment	12 week	70.03 ± 9.91*	

*p < 0.05, **p < 0.01, ***p < 0.001.

## 4 Discussion

Skin is the outermost barrier of the human body to respond to external stimuli, and its importance is self‐evident. With the aggravation of air pollution and the intensification of ultraviolet radiation, a series of chronic skin damage problems characterized by skin laxity, deepening wrinkles, and reduced moisture have been paid more and more attention. Chinese herbal medicines, derived from natural plants, animals, and minerals, have shown great potential in maintaining the skin barrier. Active ingredients such as Plaster, *Echinacea purpurea* Linn, and Dendrobium have significant effects on improving skin moisture content and protecting the skin barrier [20, 21]; collagen hydrolysates of deer sinew can be used as cosmetic materials to protect the skin from oxidative stress [22]; and the active components of sage have a certain role in inhibiting scar formation [23]. Huangbai, honeycomb, Angelica, frankincense, myrrha, and other compounds can promote the elimination of pus and the growth of new skin [24], and quercetin can stimulate skin healing after burns [25].

TCM is a living fossil inherited by the Chinese nation for thousands of years. It is an empirical medicine that relies on word of mouth. Since ancient times, TCM doctors have used drugs to cultivate kidney Yang to treat skin laxity. At the same time, “Huangdi Neijing,” the Yellow Emperor's Internal Classic, as the earliest extant medical theory book in China, also emphasizes the importance of nourishing kidney Yang to maintain skin firmness. BZBS is a kind of Chinese patent medicine, which mainly focuses on tonifying and warming kidney Yang, and also has the function of nourishing Yin. It contains Morindae Officinalis Radix, Cistanches Herba, and Epimrdii Herba, which are commonly used to nourish kidney Yang in clinical practice, with good curative effects. Animal experiments have shown that BZBS can improve the expression and circulation of MMPs, balance skin homeostasis, increase collagen content, order collagen fibers, and increase dermal thickness [26]. However, there are no reported clinical studies on BZBS to improve skin laxity, and the mechanism has not been involved. This study combined network pharmacology and clinical studies to preliminarily explore the mechanism of action of BZBS in the treatment of skin laxity.

According to our results of research, the key compounds of BZBS for skin laxity are quercetin, kaempferol, arachidonate, suchilactone, ammidin, deoxyharringtonine, sitosterol, mandenol, ethyl linolenate, stigmasterol, poriferast‐5‐en‐3beta‐ol, and cholesterol. They possibly tighten the skin by affecting the Relaxin signaling pathway, MAPK signaling pathway, PI3K‐Akt signaling pathway, JAK–STAT signaling pathway, and cellular senescence.

### 4.1 BZBS Regulates the Skin Mucosal Barrier to Improve Skin Laxity

Long‐term damage to the skin mucosal barrier caused by repeated stimulation of ultraviolet rays, insects, and ants may cause skin laxity. Arachidonate, cholesterol, and sitosterol, all of which are present in BZBS, play different roles in the skin mucosal barrier.

Arachidonate is an omega‐6 polyunsaturated fatty acid, which is a key component of the cell membrane. As lipid mediators, arachidonate and its metabolites can regulate eosinophils, mast cells, and innate lymphocytes, promote type 2 immune response, and resist parasites and allergens [27]. However, prostaglandin E2 (PGE2), a lipid‐derived signaling molecule produced by arachidonic acid through cyclooxygenase, inhibits collagen production, leading to wrinkles and impaired skin function [28, 29]. The role of arachidonate in skin barrier damage is two‐sided.

Cholesterol is an important part of sebaceous glands, constituting physical, chemical, and biological barriers of the skin, and participating in the regulation of immune function and the inflammatory process [30, 31]. Vitro studies [32] support its irreplaceable nature, showing that cholesterol works with other lipid components to prevent skin moisture loss and foreign body damage to the skin barrier.

Sitosterol is a plant sterol component with a structure similar to cholesterol, belonging to the tetracyclic triterpene class, widely found in various plant oils and nuts in nature. Our research found that sitosterol mainly plays a role in skin barrier function by anti‐inflammatory effects and improving skin lipid structure. Clinical trials have confirmed that coffee waste containing sitosterol can protect the skin by participating in oxidative stress and improve the water retention of female volunteers' skin [33]. Pannakal et al. [34] also confirmed the potential of *Filipendula ulmaria* rich in sitosterol to promote skin renewal. *Sapindus mukorossi* seed oil [35], Cariniana domestica fruit peels [36], and *Pereskia aculeata* Miller leaves [37] rich in sitosterol have shown strong anti‐inflammatory properties in both vivo and vitro experiments by resisting microbial invasion to play a role in skin barrier function. Sitosterol can also coordinate with other compounds to alter the order of skin lipid conformations and achieve high moisture retention, relieving skin dryness, and reducing the formation of fine lines [38]. Therefore, BZBS may treat skin laxity by improving skin mucosal barrier function.

### 4.2 BZBS Regulates Protein Metabolism to Improve Skin Laxity

Skin laxity is closely related to collagen loss caused by an imbalance of matrix metalloproteinases (MMPs) regulation.

In BZBS, sitosterol may not only participate in barrier protection, but also enhance collagen synthesis by reducing matrix metalloproteinases‐1 (MMP‐1) and promotes MMPs activity by deriving into hydroperoxides in response to attack by reactive oxygen species (ROS) [39]. Quercetin and kaempferol are the two most abundant components in BZBS. They are both flavonoid compounds, which are natural oxidants. Kento Takaya has demonstrated through in vitro cell experiments that quercetin combined with dasatinib can increase collagen density, increase skin elasticity, and reduce skin sagging [40]. An enzymatically modified isoquercitrin (EMIQ) containing quercetin demonstrated not only protective properties by reducing collagen damage, but also modulated both the transforming growth factor‐β (TGFβ)/Smad pathway and the MMP1 pathway, contributing to collagen preservation. This advantage was confirmed in both in vitro and clinical trials. Participants using EMIQ‐containing Essence displayed reduced facial trans‐epidermal water loss and skin roughness, alongside improved skin elasticity [41]. A clinical study related to kaempferol also supports the evidence above [42]. Using a beauty cream containing kaempferol [43] can reduce wrinkles on the skin of the participants and make the skin smoother. Other animal and clinical studies also support this idea [44, 45, 46].

The overexpression of MMP‐1 and the low expression of type I procollagen induced by ultraviolet rays are the major factors leading to wrinkles on the skin. Quercetin and kaempferol can exert the function of smoothing wrinkles by inhibiting this pathway [47, 48, 49, 50, 51]. Further examination of the upstream signaling pathways revealed that quercetin can attenuate UV‐mediated phosphorylation of extracellular signal‐regulated kinase (ERK), c‐Jun N terminal kinases (JNK), PI3K, Akt, and activator of transcription 3 (STAT3) [52, 53]. Studies on extracts containing kaempferol also confirmed that it may exhibit potent antioxidant potential and protection against skin laxity by attenuating MMP‐1 activity and collagen degradation possibly through the downregulation of MAPK/nuclear factor kappa‐B (NF‐κB)/activator protein 1 (AP‐1) signaling [54]. Combined with the results of previous studies and our study, the MAPK signaling pathway (hsa04010), P13K–AKT signaling pathway (hsa04151), and JAK–STAT signaling pathway (hsa04630) may be the one that quercetin and kaempferol regulate to tighten the skin.

## 5 Conclusion

Our study fully utilizes the bridge role of network pharmacology in displaying the relationship between TCM active compounds and disease targets, and for the first time, conducts a preliminary exploration of the potential of BZBS in treating skin laxity. It is found that the representation of key compounds, including quercetin, kaempferol, cholesterol, and sitosterol, shows outstanding advantages in improving wrinkle depth, skin elasticity, and moisture content. Our work also explains the mechanisms of BZBS in treating skin laxity from the perspectives of repairing the skin mucosal barrier and regulating protein metabolism. Although our study provides novel insights into BZBS's antiaging potential, certain aspects merit consideration in future research. The clinical observations, though promising, were derived from a modest cohort of female volunteers over a 12‐week observation period. Although network pharmacology revealed biologically plausible targets and pathways (e.g., PI3K‐Akt signaling, collagen regulation), these computational predictions would be enriched by subsequent cellular‐level validation. The inclusion of a comparator group in future research could further elucidate intervention‐specific effects. Environmental standardization protocols may also enhance precision in dermatological parameter measurements. These observations collectively highlight opportunities for refining study designs in subsequent investigations of herbal antiaging formulations. We hope the results may provide some guidance for more rigorous pharmacological and clinical research in the future.

## Author Contributions

Cuicui Cheng and Jun Mei contributed to the review and editing of the manuscript. Fengqin Xu provided conceptualization support. Yuanbai Li and Yang Yang curated and analyzed data. Limin Yang supervised the research process. Jing Li and Xiaojie Zhang participated in data checking and interpretation of data results. Fangzhou Liu and Meng Hong provided guidance for the network pharmacology and clinical research design, revised the manuscript, and ensured the overall integrity of the experimental design. Zhenhua Jia oversaw the entire clinical data analysis process, ensuring methodological rigor and scientific validity in data interpretation. Second, during manuscript revisions, they conducted rigorous multi‐round evaluations of statistical results, refining analytical frameworks to strengthen the accuracy and clarity of research conclusions. All personnel participated in discussions of the experimental results and contributed to the final manuscript.

## Ethics Statement

This study was approved by the Ethics Committee of Xiyuan Hospital (Approval Number: 2022XLA034‐2). It followed Good Clinical Practice (GCP) guidelines and the Declaration of Helsinki.

## Conflicts of Interest

The authors declare no conflicts of interest.

## Data Availability

The data that support the findings of this study are available from the corresponding author, upon reasonable request.
